# Changing Epidemiology of *Candida* spp. Causing Bloodstream Infections in a Tertiary Hospital in Northern Greece: Appearance of *Candida auris*

**DOI:** 10.3390/pathogens14020161

**Published:** 2025-02-07

**Authors:** Athina Pyrpasopoulou, Charalampos Zarras, Eleni Mouloudi, Georgios Vakalis, Argyro Ftergioti, Dimitrios Kouroupis, Anastasia-Izampella Papathanasiou, Elias Iosifidis, Stella Goumperi, Charis Lampada, Maria Terzaki, Emmanuel Roilides

**Affiliations:** 1 2nd Propedeutic Department of Internal Medicine, Aristotle University of Thessaloniki, Hippokration General Hospital, 54642 Thessaloniki, Greece; dimcour841@gmail.com (D.K.); mary.terz@hotmail.com (M.T.); 2 Infectious Diseases Unit, Hippokration General Hospital, 54642 Thessaloniki, Greece; giwrgosvakalis1998@gmail.com (G.V.); arofter@gmail.com (A.F.); anizelidi@yahoo.gr (A.-I.P.); stellagoumperi@gmail.com (S.G.); roilides@gmail.com (E.R.); 3 Laboratory of Microbiology, Hippokration General Hospital, 54642 Thessaloniki, Greece; zarraschak6@gmail.com; 4 Intensive Care Unit, Hippokration General Hospital, 54642 Thessaloniki, Greece; elmoulou@yahoo.gr; 5 3rd Department of Pediatrics, Aristotle University of Thessaloniki, Hippokration General Hospital, 54642 Thessaloniki, Greece; iosifidish@gmail.com (E.I.); hlampada@hotmail.com (C.L.)

**Keywords:** candidemia, epidemiology, *C. auris*, resistance

## Abstract

Introduction: The epidemiology of candidemia has shifted in the past few decades; drug-resistant *non-albicans Candida* species have become more prevalent worldwide. The aim of this retrospective study was to determine the epidemiology of *Candida* species isolated from hospitalized neonates, children and adults, and to investigate a potential changing susceptibility pattern in a large general tertiary hospital. Methods: All unique *Candida* strains isolated from candidemia cases between 1 January 2020 and 15 October 2024 were identified, and their susceptibility profile was characterized. The distribution pattern in different ward types (medical, surgical, pediatric and ICU) was recorded. Cumulative annual susceptibility profiles were compared. Results: Candidemia incidence increased during the COVID-19 pandemic, from 0.63/1000 patient-days in 2020 to 0.96/1000 patient-days in 2022, and has since slightly decreased (0.83 and 0.89 in 2023 and 2024, respectively). Candidemia-associated mortality was high (>50%) in 2020 and peaked during the pandemic. During the study period, *Candida parapsilosis* remained the most frequent *Candida* spp. However, since the first isolation of *Candida auris* from the bloodstream in late 2022, and despite intense infection control measures taken, its frequency sharply climbed to the second position after only *C. parapsilosis* in the first 10 months of 2024 (33.6% vs. 25.2% for *C. parapsilosis* and 21.0% for *C. albicans*). While *C. albicans* has remained highly susceptible to fluconazole (1% resistance rate), *C. parapsilosis* manifested significant resistance to fluconazole during 2022–2024 (52%). *C. auris* was universally resistant to azoles and one isolate also resistant to echinocandins. Conclusions: A high prevalence of azole resistance of *C. parapsilosis,* the most frequently isolated *Candida* species, persists, and a significant rise of *C. auris* was recorded in nosocomial bloodstream infections with severe implications on public health.

## 1. Introduction

Candidemia represents one of the most frequent causes of healthcare-associated bloodstream infections [1]. It ranks roughly fourth in frequency in ICUs depending on the profile of the patients, the primary site of infection, co-morbidities and risk factors predisposing to the development of fungemia [2,3]; however, it is still considered an opportunistic infection. *Candida* spp. are common commensals of the skin and mucosal surfaces of healthy humans but can become pathogenic when normal flora is disrupted, either by broad spectrum antibiotics, by immunosuppression (mainly prolonged neutropenia and steroid use), and by breaching of mucocutaneous barriers, i.e., with the insertion of indwelling catheters [4]. It affects mainly the neonatal and immunocompromised, such as the elderly population, and impacts patients’ cost of treatment and prognosis significantly, as it causally prolongs patients’ hospitalization, and is associated with severe mortality, which may even exceed 70% with new emerging drug-resistant species [5,6].

*Candida albicans* remains the most prominent species isolated from blood; in children more readily so than in the adult population [4]. However, a global shift from *C. albicans* to *non-albicans* species has become obvious already in the last two decades [7]. *Candida albicans* nowadays is isolated in <50% of invasive *Candida* infections; this could, but only in part, be explained by the rising incidence of candidemia in the elderly. Although the trend towards *non-albicans* species is universal, epidemiology varies considerably with different studies reporting a rise in *Candida parapsilosis*, *Candida tropicalis* and/or *Candida glabrata* strains [8]. The changing epidemiology towards *non-albicans Candida* species is of particular concern, as these are usually also characterized by significant resistance to first-line antifungal agents [9]. Progressively, since its first official identification in 2009, the highly transmissible and azole-resistant *Candida auris* appears to significantly aggravate the global healthcare-associated infection burden [10].

The aim of this retrospective study was to determine the most frequently isolated *Candida* species from hospitalized children and adults in the last five years, and to investigate a potential changing susceptibility pattern in a large general tertiary hospital.

## 2. Materials

All unique *Candida* strains isolated from candidemia cases between 1 January 2020 and 15 October 2024 in Hippokration General Hospital, in Thessaloniki, Northern Greece, were included in the analysis. Hippokration General Hospital features 800 beds, 4 Intensive Care Units (1 multivalent for adults, 1 for children and 2 for neonates), 1 Solid Organ Transplantation Unit, and regularly followed-up chronic immunosuppressed patients (dialysis, oncology and hematology patients). Repeat candidemia episodes in the same patient were excluded. The causative strains were identified by Vitek 2 (bioMérieux, Marcy l’Etoile, France), and as of the beginning of 2024 were confirmed additionally by MALDI-TOF, and their susceptibility profile was characterized by Vitek 2 (bioMérieux). EUCAST susceptibility cut-offs were used for isolate characterization [11]. In the cases of *C. auris*, isolates’ susceptibility testing was performed by Sensititre YeastOne assay (Thermo Fisher Scientific, Waltham, MA, USA). Resistance rates to antifungals were determined and cumulative annual susceptibility profiles were compared.

The incidence of candidemia in different ward types (medical, surgical, pediatric and ICU) was recorded. The outcome was associated with the type of *Candida* spp. (*albicans* vs. *non-albicans*). This study was conducted in accordance with the Declaration of Helsinki and approved by the local ethics committee (Approval No.: 53205/29-11-2024, FF:24-E-82).

## 3. Results

**Figure 1:**
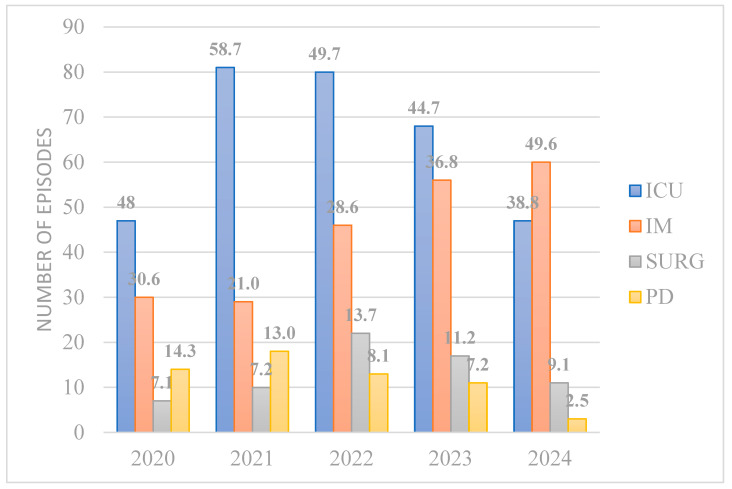
Distribution of recorded candidemia episodes per ward type. ICU: Intensive Care Unit; IM: Internal Medicine; SURG: surgical wards; PD: pediatric wards, pediatric ICU and neonatal ICU. Numbers above bars represent percentages of number of candidemia cases in each ward type per total annual number of candidemia episodes.

**Figure 2:**
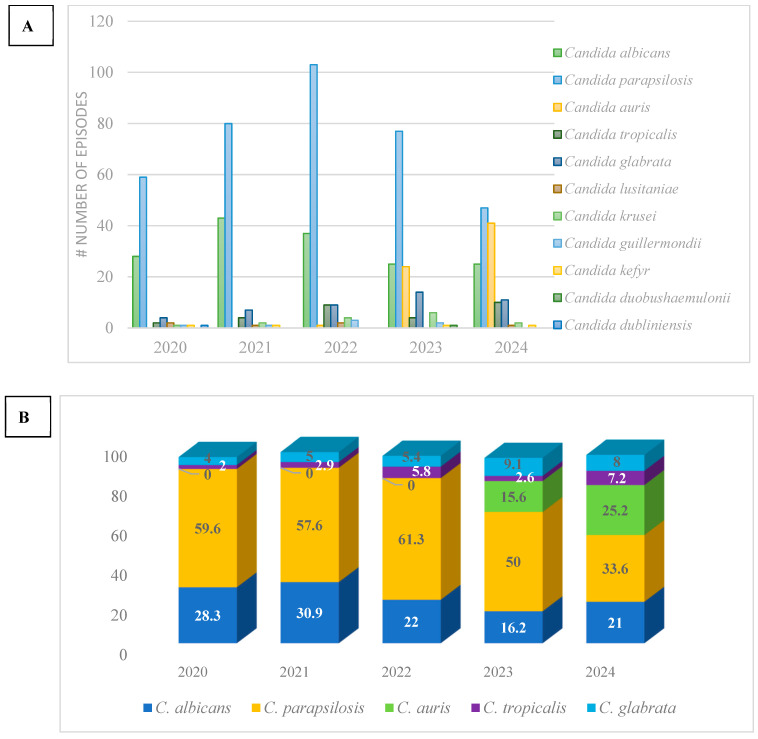
(**A**). Annual distribution of *Candida* spp. isolates from *Candida*-positive bloodstream infections during 2020–2024 period. (**B**). Relative contribution of *Candida* spp. isolates in annual distribution of candidemia.

A total of 657 unique episodes of candidemia were included in this retrospective analysis from 1 January 2020 to 15 October 2024 (99 in 2020, 139 in 2021, 168 in 2022, 154 in 2023 and 119 in 2024, respectively). In total, 14 pediatric vs. 84 adult patients in 2020 developed fungemia, 18 vs. 120 in 2021, 13 vs. 148 in 2022, 11 vs. 141 in 2023, and 3 vs. 115 in 2024, respectively. The incidence of candidemia rose during the pandemic from 0.63/1000 pt-days in 2020 to 0.96/1000 pt-days in 2022 and has since slightly decreased (0.83 and 0.89 in 2023 and 2024, respectively). It needs to be pointed out that the duration of hospital stay was higher during the pandemic, largely attributed to the increased admission rates in the ICUs. Until 2023, *Candida* bloodstream infections were more common among critically ill patients. In 2024, 60/121 (49.6%) of candidemia episodes were recorded in medical wards (Figure 1). *C. albicans* has lost the lead with a relative contribution in the total recorded cases of 28/99 (28.3%) in 2020, 43/139 (30.9%) in 2021, 37/168 (22.0%) in 2022, 25/154 (16.2%) in 2023 and 25/119 (21.0%) in 2024. *C. parapsilosis* has instead risen to the first place and has become the most frequently isolated species from *Candida* bloodstream infections, with a relative contribution of 59/119 (59.6%) in 2020, 80/139 (57.6%) in 2021, 103/168 (61.3%) in 2022, 77/154 (50.0%) in 2023 and 40/119 (33.6%) in 2024 throughout the period of the study. An alarming rise in *C. auris* implication in invasive *Candida* infections has been noted. Since its first isolation in 2022, *C. auris* has been identified as the implicated pathogen in 25/154 (15.6%) of candidemia cases in 2023 and 30/119 (25.2%) in 2024, respectively. Analytical distribution of all isolated *Candida* spp. and their relative contribution in invasive bloodstream infections per annum is described in Figure 2A,B. Death was recorded in 58.4% of candidemia cases in 2020, 76.1% in 2021, 79.3% in 2022, 79.4% in 2023 and 71.3% in 2024. Similar mortality rates were noted between *albicans* and *non-albicans* candidemia (57.7 vs. 58.7% in 2020, 78.6 vs. 75% in 2021, 88.6 vs. 75.9% in 2022, 81.8 vs. 78.9% in 2023 and 65.2 vs. 71.8%, respectively).

Data on resistance to broadly used antifungals were analyzed from 1 January 2022 to 15 October 2024. *C. albicans* remained susceptible to fluconazole; 1 strain out of 79 was found to be resistant. However, more than half (52%) of *C. parapsilosis* were resistant to fluconazole, which is a significantly higher value than that previously reported in national data (Candida III). All *C. auris* isolates were resistant to fluconazole; only one isolate was additionally resistant to echinocandins (see Table 1).

## 4. Discussion

Candidemia is a serious complication of critical illness leading inpatients to prolongation of hospitalization and to unfavorable outcomes by increasing mortality to 50–70% or even higher. Several factors related to the patients’ profile or their clinical course and management have been associated with the development of invasive *Candida* disease [12]. According to published reports, incidence rates are higher among children and the elderly, vary geographically, and average 0.3–0.5/1000 patient-days [2]. In the last two decades, an increase in the incidence of invasive *Candida* infections has been described [13], potentially attributed to the advances in the development and application of immunosuppressive/immunomodulatory treatments. The pandemic of COVID-19 aggravated this phenomenon further, by leading to an overflow of critically ill patients admitted in ICUs [14,15,16], and the large-scale administration of corticosteroids and immunosuppressive treatments rather than the overuse of antifungal prophylaxis leading to breakthrough infections. Current guidelines for immunosuppressed patients have revisited the concept of broadly applied antifungal prophylaxis, and the predominance of *non-C. albicans* species, potentially resistant to first-line azoles, has put aside their use, which could drive the predilection of *C. auris*. Moreover, the outcome of patients with candidemia has also worsened. In epidemiological studies, COVID-19-positive patients with candidemia had significantly worse clinical outcomes than others, probably as an effect of their immune dysregulation, medical treatment, implantation of devices and prolonged mechanical ventilation. In agreement with similar observations, our retrospective epidemiological study shows an increase in recorded candidemia cases through the severe pandemic waves; incidence rose to 0.96/1000 patient-days in 2022 from 0.63/1000 patient-days recorded in 2020, respectively. Candidemia-associated mortality was high throughout (>50%). During the pandemic, it peaked, reaching almost 80%, regardless of the *Candida* spp. implicated in the infection and has since remained higher than 70%.

The ubiquitously reported change in *Candida* spp. epidemiology in isolates from invasive infections from *albicans* to *non-albicans* was also noted in our study [4,16,17]. The relative reduction in the contribution of *C. albicans* in systemic *Candida* infections became more established over the years. Instead, *C. parapsilosis* prevailed, becoming the most prevalent species (61.3% of all candidemias in 2022). Although *C. albicans* remained susceptible to widely used first-line antifungals, *C. parapsilosis* has become increasingly more resistant to fluconazole (52%), potentially related to prolonged treatment with antifungal prophylaxis, especially in high-risk inpatients. Although in the last two years of the study, and in the aftermath of the pandemic, a tendency for invasive *Candida* infections to lessen could be identified, alarmingly, the “new kid on the block”, *C. auris*, made its appearance in late 2022, and has since settled in the environment with an exponentially rising contribution in nosocomial invasive fungal infections (15.6% in 2023 rising to 25.2% in 2024). Although data for 2024 have not been finalized yet, cases of *C. auris* infection reach up to 15.10 as the second most frequently isolated *Candida* spp. from bloodstream infections at present. Since the first *C. auris*-associated candidemia case was reported in January 2023, strict healthcare policies and infection control measures have been implemented to halt the nosocomial spread [18]. Unfortunately, its features as a pathogen allow *C. auris* to spread rapidly, colonize patients and their personal environment and firmly settle itself in the nosocomial establishment. This phenomenon could at least to some extent be also attributed to the increased admission rates and hospital stays during the pandemic and the widespread use of immunosuppressants during this period. Health practices, environmental factors, climate change, virulence factors, etc., perhaps influence the increase in *C. auris* in Greece. As we are gradually transitioning to more difficult-to-treat fungi, such as the azole- and potentially echinocandin-resistant *C. auris* [19], emphasis should be particularly placed on the early recognition and elimination of risk factors leading to the development of candidemia and invasive fungal infections in general to safeguard strict surveillance programs, and to apply stringent infection control programs to help contain and potentially reverse this phenomenon.

## 5. Conclusions

This study documented the high prevalence of azole resistance of *C. parapsilosis*, the most frequently isolated *Candida* species, and a significant rise in *C. auris* in nosocomial bloodstream infections with seriouss implications for the management of hospitalized patients and public health.

## Figures and Tables

**Table 1:** *Candida* spp. antimicrobial resistance rate (1 January 2022 to 15 October 2024).

	*C. albicans* (79)		*C. glabrata* (32)		*C. parapsilosis* (249)		*C. kefyr* (2)		*C. tropicalis* (26)		*C. krusei* (11)		*C. lusitaniae* (4)	
	R (%)	Total AST	R (s%)	Total AST	R (%)	Total AST	R (%)	Total AST	R (%)	Total AST	R (%)	Total AST	R (%)	Total AST
Fluconazole	1%	79			52%	249			4%	26	100%	11		
Voriconazole	4%	79	0%	1	17%	248			0%	26				
Caspofungin	0%	79	0%	31	0%	247	0%	2	0%	26	0%	11	0%	3
Micafungin	1%	79	3%	32	1%	249			0%	1				
Amphotericin B	3%	79	0%	32	2%	248			0%	26	18%	11		
Flucytosine	3%	76	0%	31	1%	242	0%	2	4%	26	0%	11	25%	4
Total	2%	471	1%	127	12%	1243	0%	4	2%	131	30%	44	14%	7

## Data Availability

The original contributions presented in this study are included in the article. Further inquiries can be directed to the corresponding author.
